# Effect of Ribavirin Therapy on Salivary Gland Function: An Oral Medicine Prespective

**DOI:** 10.5812/kowsar.1735143X.786

**Published:** 2011-11-30

**Authors:** Nima Mahboobi, Zahra Haghighi

**Affiliations:** 1 Department of Oral and Maxillofacial Surgery, Faculty of Dentistry, Tehran University of Medical Sciences, Tehran, Iran; 2 Students’ Scientific Research Center, Tehran University of Medical Sciences, Tehran, Iran; 3 Department of Gastroenterology and Liver Diseases, Faculty of Medicine, Tehran University of Medical Sciences, Tehran, Iran

**Keywords:** Ribavirin, Salivary Gland, Oral Medicine, Sialadenitis, Hepatitis C

Dear Editor,

We read with keen interest the recently published paper entitled "Ribavirin impairs salivary gland function during combination treatment with pegylated interferon alfa-2a in hepatitis C patients" by Aghemo et al[1] in your esteemed journal. The study described the effect of ribavirin plus interferon therapy (combination therapy) on salivary gland function. However, we think we must put pen to paper with respect to draw the attention of your respectful readers to possible misinterpretation of the data that has been presented.

Even at the beginning of the third millennium, hepatitis C virus (HCV) infection remains a major global public health problem, and to date, combination therapy seems to be the most effective treatment option. Nonetheless, this treatment might result in some side effects such as lichen planus, skin rash, psychological disturbances, poor appetite, anemia, and leucopenia [2].

Derived results of the study by Aghemo et al might be affected by some confounding factors. First, although the authors had mentioned that a limitation of their study was using patients who had hepatitis B virus (HBV) infection as the control subjects, it cannot iron out all the attributed results of choosing an unsuitable control group. Previous studies have shown that HCV infection in the epithelial cells of the salivary glands is a factor associated with the development of sialadenitis and Sjogren's syndrome [3]. However, to our knowledge, no previous study has shown such a conclusive correlation with HBV infection. Because of the observed inconsistencies in the findings of these studies, it does not seem appropriate to conclude that the use of drugs is the sole cause of salivary gland malfunction. In addition, it is unclear that why the authors used such a small control group; the use of a small sample size raises questions regarding the reliability of the rate of side effects expressed by the patients in this group.

An advantage of their study was that they used stimulated saliva for evaluating sialadenitis. Previous studies have confirmed that stimulated whole saliva is especially useful in measuring salivary gland function in patients with common autoimmune disorders. The main advantage of gustatory stimulation is reduction in the variability of salivary flow rates among study subjects [4]. Several studies have been conducted on the role of oral fluids (namely saliva and gingival crevicular fluid) in viral hepatitis. However, no conclusive results have been obtained on the role of oral fluids in the transmission or diagnosis of viral hepatitis, its progress, or treatment monitoring.

Further studies are required to assess the variations in the levels of oral fluid secretion in patients with viral hepatitis. If such changes can be proven, it might help identify why the rate of dental caries is significantly higher in patients with HCV infection than in the general population [5].

We suggest that randomized controlled trials and experimental observations, including salivary gland function monitoring before and after treatment, to be conducted in HCV-infected patients. Evaluating the presence of HCV in salivary gland tissues could also yield accurate results.
